# Macrophage Phenotypes in Normal and Diabetic Wound Healing and Therapeutic Interventions

**DOI:** 10.3390/cells11152430

**Published:** 2022-08-05

**Authors:** Hadeel Al Sadoun

**Affiliations:** 1 Department of Medical Laboratory Technology, Faculty of Applied Medical Sciences, King Abdulaziz University, Jeddah 21589, Saudi Arabia; hsadounkau.ed.sa@kau.edu.sa; Tel.: +966-(12)-6400000 (ext. 24277); 2 Stem Cell Unit, King Fahad Medical Research Centre, King Abdulaziz University, Jeddah 21589, Saudi Arabia

**Keywords:** tissue regeneration, tissue remodeling, inflammation, differentiation, macrophage polarization

## Abstract

Macrophage differentiation and polarization are essential players in the success of the wound-healing process. Acute simple wounds progress from inflammation to proliferation/regeneration and, finally, to remodeling. In injured skin, macrophages either reside in the epithelium or are recruited from monocytes. Their main role is supported by their plasticity, which allows them to adopt different phenotypic states, such as the M1-inflammatory state, in which they produce TNF and NO, and the M2-reparative state, in which they resolve inflammation and exhibit a reparative function. Reparative macrophages are an essential source of growth factors such as TGF-β and VEGF and are not found in nonhealing wounds. This review discusses the differences between macrophage phenotypes in vitro and in vivo, how macrophages originate, and how they cross-communicate with other cellular components in a wound. This review also highlights the dysregulation of macrophages that occurs in nonhealing versus overhealing wounds and fibrosis. Then, the therapeutic manipulation of macrophages is presented as an attractive strategy for promoting healing through the secretion of growth factors for angiogenesis, keratinocyte migration, and collagen production. Finally, Hoxa3 overexpression is discussed as an example of the therapeutic repolarization of macrophages to the normal maturation state and phenotype with better healing outcomes.

## 1. Introduction

When the skin barrier is breached, wound healing follows a well-regulated process that requires interplay between keratinocytes, inflammatory cells, and endothelial cells and is regulated by cytokines, growth factors, and transcription factors. This dynamic and complex process is normally simplified into four distinct but overlapping phases: hemostasis, inflammation, cell migration and proliferation, and tissue regeneration and remodeling [1].

Most of our understanding of the molecular biology and mechanisms of the wound-healing process and the relative time course needed for healing to take place originated from studies using mouse models. Therefore, we need to consider that healing in humans may differ with regard to the time needed for the repair process, as human skin regenerates at a much slower rate, ultimately by a re-epithelialization process, whereas, in rodents, the contractile ability of the panniculus carnosus shortens the time span necessary for wound resolution [2].

The outcome of wound healing varies dramatically according to factors such as the age and health status of the patient and the site and severity of the wound [3]. Prenatal wounds, for example, can heal perfectly without scar tissue formation [4,5], whereas patients with diabetes mellitus (DM) suffer from chronic wounds that fail to heal within the standard timeframe and can evolve into diabetic ulcers, ultimately leading to the risk of limb amputation [6]. Excessive healing, on the other hand, refers to the overaccumulation of pathological connective tissue, forming hypertrophic scars and keloids [7]. These forms of tissue fibrosis can be very challenging for patients and clinicians, particularly when they occur at a skin area where flexibility is needed, such as a joint [7]. The mechanisms underlying the variable outcomes of healing, from nonhealing in DM to overhealing in fibrosis, are not completely understood. Common pathways show the involvement of inflammatory cells, particularly macrophages, and show how they can direct the healing outcome toward regeneration or fibrosis [8,9,10]. Therefore, the role of macrophages in healing and tissue regeneration has attracted considerable attention, leading to the accumulation of evidences elucidating their pivotal role in each phase of normal healing. Once their role is disturbed, prolonged inflammation and pathological healing may occur, as observed in chronic wounds.

This review first covers the regulation of each phase of wound healing, focusing on the role of macrophages, before illustrating how macrophage phenotypes are described in the literature. A model of macrophage phenotypes that compares their effects in normal wounds and their defects in diabetic wounds is then proposed. Finally, some examples of therapeutic approaches to treating diabetic wounds, such as limiting inflammation and inducing tissue regeneration through the therapeutic manipulation of macrophages, are discussed.

## 2. Wound Healing

### 2.1. Hemostasis

**Figure 1:**
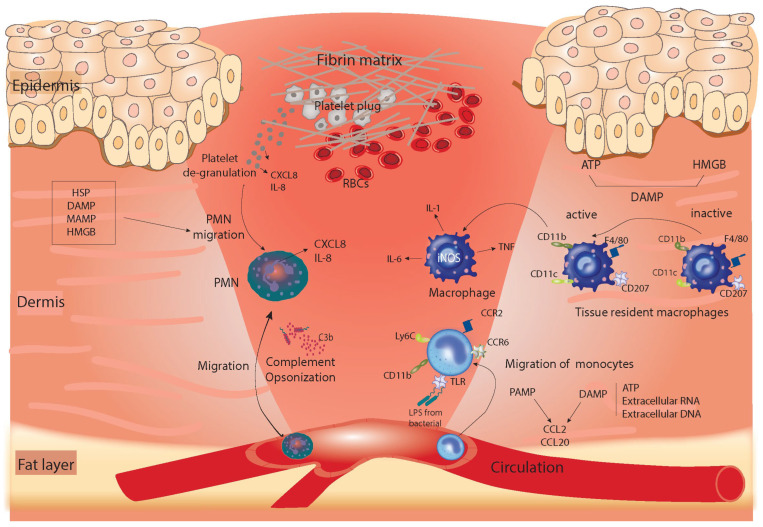
Summary of the coagulation and inflammation phase of healing. The repair process begins with the formation of fibrin-based clots and the inflammatory response. Neutrophils were recruited in response to products released from platelet degranulation releasing IL-8 and CXCL8 and also by chemotactic signals produced from DAMP, MAMP, HSP, and HMGB and by complement opsonization. Monocytes migrate later in this phase from the circulation into the tissue due to LPS produced by invading bacteria, which are bound to TLR on the surface of monocytes. This pathway forms one example of the PAMP-stimulated migration of monocytes. Products released from damaged tissue such as ATP, extracellular RNA, or DNA influence monocyte migration. PAMP- or DAMP-mediated monocyte recruitment can stimulate interleukins or chemokines such as CCL20 and CCL2, which bind to their receptors—CCR6 and CCR2, respectively—on monocytes. Wound resident macrophages also play a role in this phase as they are activated by DAMP, including ATP and HMGB. ATP, adenosine triphosphate; DAMP, damage-associated molecular pattern; HMGB, high-mobility group box protein; LPS, lipopolysaccharides; PMN, polymorphonuclear neutrophil; PAMP, pathogen-associated molecular pattern; TLR, Toll-like receptors; HSP, heat shock protein; MAMP, microbes-associated molecular patterns.

The very first event following injury is the activation of hemostasis. This includes both pathways of coagulation: the extrinsic and intrinsic pathways. In the injured area of the skin, when blood leaks into the exposed collagen, the release of potent vasoconstrictors, such as serotonin, is stimulated [1]. Platelets aggregate in the exposed collagen to form a temporary plug embedded in the cross-linked fibrin matrix formed at the end of the coagulation cascade. The fibrin-based plug not only serves to stop the hemorrhage but also acts as a scaffold to which inflammatory cells and growth factors can bind [11]. Activated platelets serve as the earliest provider of growth factors, such as platelet-derived growth factor (PDGF), transforming growth factor (TGF)-β, and TGFα, which prime the wound for the inflammatory phase of healing and lead to the passive leakage of polymorphonuclear (PMN) neutrophils from the damaged blood vessels into the wound (Figure 1) [11]. TGFβ production is induced in several waves during skin injury, with the first wave produced from platelets in the proinflammatory phase. This stimulates the chemoattraction of innate immune cells, such as neutrophils and monocytes [12,13,14,15]. Platelet degranulation leads to the release of inflammatory chemoattractants, including IL-8 or CXCL8 as well as TNF, IL-1β, and IL-1α. Platelet degranulation also activates a complement cascade, leading to the production of the cleaved product C5a by the C5 convertase enzyme, which is a potent neutrophil chemotactic protein [1,16,17,18]. The presence of microorganisms further stimulates the activation of the alternative complement pathway. C3b mediates bacterial opsonization, and this further stimulates neutrophil recruitment to the wound site [1,16,17] (Figure 1). By the end of the hemostasis phase, the activated complement cascade triggers mast cells to releases histamine, which causes the expansion of the capillaries and the further recruitment of inflammatory cells into the wound [16,19,20]. This forms the basis of the transition from hemostasis to the inflammatory phase of healing. Altogether, the above knowledge emphasizes the roles that platelets play in communication with other cells and in the recruitment of inflammatory cells to initiate the next phase of healing.

### 2.2. Inflammatory Phase

The inflammatory phase largely overlaps with hemostasis and occurs within the first 72 h of wound healing. It is best described as an interplay between the following components: regulatory signals, leukocyte infiltration, the control of tissue damage, and the removal of debris/pathogens from the wound [16]. Neutrophils are the first cells to infiltrate the wound from the circulation to sterilize the injured area [21]. This process is facilitated by the activation of endothelial cells that express P- and E-selectin molecules, which, in turn, allows the cells to roll along the vessel wall and extravasate from the circulation into the wound tissue [22]. At the damaged tissue, neutrophils are recruited in response to growth factors released during platelet degranulation as well as chemotactic signals from the damage-associated molecular patterns (DAMPs), microbe-associated molecular patterns (MAMPs), heat shock proteins (HSPs), and high-mobility group box proteins (HMGBs) [23,24,25]. Neutrophils themselves act as chemoattractants by releasing additional IL-8 and CXCL8, which are recognized by tissue-resident cells, including macrophages, dendritic cells, and the endothelium, creating local cell activation. The activation of neutrophils is facilitated by signals from inflammatory cytokines, such as interleukin-6 (IL-6), IL-1α, IL-1β, and tumor necrosis factor (TNF) [25].

**Figure 2:**
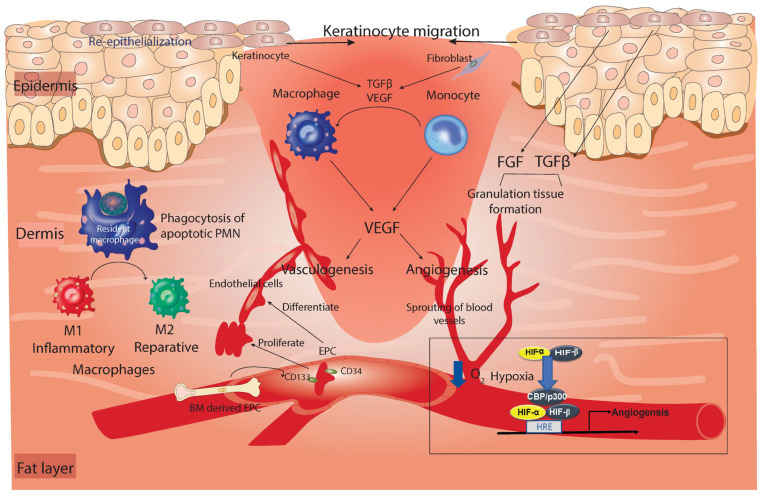
Summary of the late inflammation and proliferation phase of healing. Keratinocytes migrate to the wound and proliferate around the outermost area of the wound to close the wound surface by re-epithelialization. Underneath the keratinocyte layer, granulation tissue is formed and replaces the damaged dermis in a process that depends on growth factors such as FGF and TGF-β. Macrophages are differentiated from monocytes in response to TGF-β and VEGF growth factors released from keratinocytes and fibroblasts. In addition, during this phase, resident macrophages (purple-colored in the dermis) are directly involved in the elimination of inflammatory cells from the wound by engulfing apoptotic PMNs (efferocytosis). This process contributes to the switch of the macrophage’s phenotype from M1 to M2. The damaged blood vessels are replaced by neovascularization through angiogenesis, in which new blood vessels sprout from healthy vessels regulated via alterations in the oxygen gradient through HIF. Alternatively, it can form via vasculogenesis, in which endothelial cells are combined to create a new branch of the blood vessel in a process that requires EPC migration from the BM. VEGF produced from reparative macrophages or fibroblasts stimulates both processes of neovascularization. TGF-β, transforming growth factor beta; VEGF, vascular endothelial growth factor; FGF, fibroblast growth factor; HIF, hypoxia-inducible factor; CBP/p300, core-binding factor/protein 300; HRE, hypoxia response element.

Excessive neutrophil involvement in a wound can adversely affect tissue and slow down healing. This is evident from recent studies on human diabetic ulcers and streptozotocin-induced diabetic murine models that were primed for increased NETosis, the process that forms extracellular neutrophil traps [26]. Following the induction of angiogenesis, during the mid-stage of healing, neutrophils can reverse transmigrate out of the tissue and back into the blood vessels. Alternatively, neutrophils commit suicide and are eaten by macrophages [21] (see Figure 2). Inflammation is well controlled by the release of growth factors, particularly TGF-β, which is known for its paradoxical effect in wound healing. It was first described as an anti-inflammatory growth factor, because TGFβ^null^ mice die a few days after birth due to excessive inflammation that reaches vital organs such as the lungs and skin [12,14,27]. However, TGF-β was later described as a proinflammatory mediator by acting as a chemoattractant of inflammatory and endothelial cells as well as by contributing to the production of the IL-1β, TNF, and IL-6 inflammatory cytokines and macrophage differentiation [14,28]. Altogether, this gave TGF-β the reputation of being a double-edged sword that can work differently depending on the stage of healing and the context.

Monocytes migrate later during the inflammatory process through direct involvement in the phagocytic immune response or by differentiating into inflammatory macrophages [29]. Monocyte-derived macrophages differ from conventional dendritic cells that reside in the skin epidermis, termed Langerhans cells (LCs), or cells in the skin dermis, termed dermal macrophages [30]. LCs express surface markers, such as langerin, CD207, MHCII (highly), and CD11c, which differentiates them from the dermal macrophage population [30]. The specific role of LCs during the healing process remains unclear. However, evidence indicates that the number of LCs increases in the epidermal layer during the early phases of healing and during the healing of diabetic foot ulcers (DFUs) compared to in nonhealing DFUs, suggesting a possible therapeutic role of LCs in diabetic wounds [31].

Studies that aimed to test the functions of other immune cell responses throughout the healing process have provided clues on the role of each leukocyte subpopulation within the wound. Compared with monocytes, macrophages can play more diverse roles and can take over the phagocytic activity of PMNs and monocytes, owing to their plasticity, further permitting their transition from inflammatory (during the early stages of healing) to anti-inflammatory/reparative (during the proliferative stage) [32,33].

The origin of macrophages within the wound is either tissue-resident macrophages (CCR2^lo^, Cx3CR1^hi^) that form a small proportion of macrophages within the wound or, more frequently, the monocyte-derived population (CCR2^hi^, CX3CR1^lo^), which can increase in number in response to inflammatory stimuli (reviewed in [34]). The presence of macrophages, particularly the inflammatory type, is controlled mainly by proinflammatory mediators and chemokines, including RANTES/CCL5 and monocyte chemotactic protein-1 (MCP-1), which are released from platelet degranulation, fibrin clots, keratinocytes, fibroblasts, and leukocytes themselves [35]. At the end of the inflammatory phase, macrophages are the dominant cell type in the wound and maintain antimicrobial activity through the production of IL-1, IL-6, TNF, and inducible nitric oxide synthase (iNOS) [32,33]. Figure 1 summarizes the main changes that occur in the skin during coagulation and the early inflammatory phase of healing.

### 2.3. Proliferation and Tissue-Regeneration Phase of Healing

This phase comprises cellular proliferation, migration, and the active repair of damaged blood vessels and dermis. Morphologically, the damaged dermis is replaced by granulation tissue, which is composed of invading capillaries that give it a granular appearance [1]. The repair of the damaged blood vessels can occur through two mechanisms: angiogenesis and vasculogenesis [36]. In angiogenesis, blood vessels sprout from intact capillaries to replace the damaged vessels in a process that is regulated by alterations in the oxygen gradient. Hypoxia-inducible factor (HIF) is the master regulator of this process. During hypoxic conditions, the HIF-α subunit dimerizes with the HIF-β subunit, and the complex binds to the hypoxia response element (HRE) on the target genes in a process that requires the presence of the co-factor, core-binding factor (CBP)/ protein 300 (p300), thus activating a panel of genes needed for vascular growth and angiogenesis (Figure 2) [37]. In vasculogenesis, endothelial progenitor cells (EPC) expressing CD34 and CD133 markers for early EPCs, together with VEGFR, are released from the BM into the circulation in which they migrate to sites of vasculogenesis. In those sites, they can proliferate or differentiate into endothelial cells and form aggregates of endothelial cells independent of existing capillaries (Figure 2) [36]. The most important regulator of both neovascularization processes is the macrophage-derived vascular endothelial growth factor (VEGF), which switches to epidermal-derived VEGF during the late stages of tissue repair [38]. Other sources that produce VEGF growth factors are keratinocytes, fibroblasts, and mast cells [39,40].

**Figure 3:**
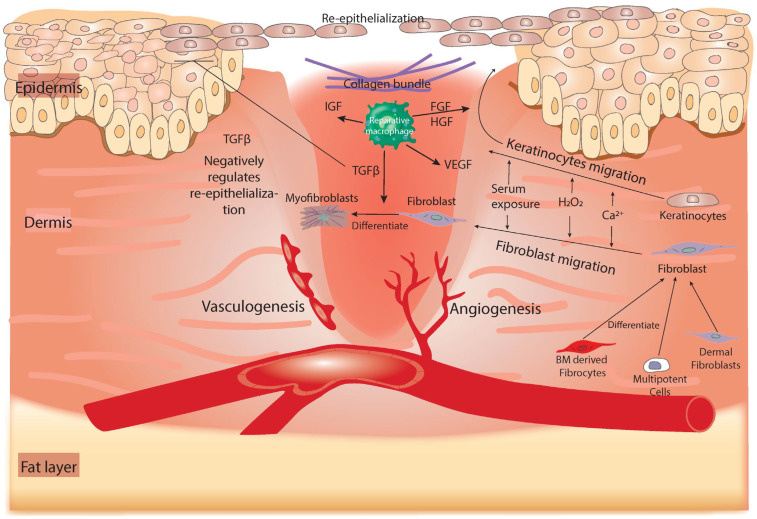
Summary of the tissue-regeneration phase of healing. In this phase, re-epithelialization and the formation of collagen-based ECM occur. Keratinocytes and fibroblasts can migrate to the wound in response to releasing H_2_O_2_, exposed Ca2+, and serum from damaged tissue. Fibroblasts are provided to the injured tissue either from the healthy dermis, BM-derived fibrocytes, or multipotent precursor cells. Both fibrocytes and multipotent cells can differentiate into fibroblasts before they are recruited to the wound. Reparative macrophages (green-colored in the wound) play an essential role in this phase by producing several growth factors, including FGF, TGF-β, IGF, and VEGF. TGF-β promotes the differentiation of fibroblasts into myofibroblasts, a type of contractile cell that can re-approximate the wound edges. Additionally, it can negatively regulate the re-epithelialization process. FGF and HGF are associated with keratinocyte migration to the wound edge to perform re-epithelialization. BM, bone marrow; FGF, fibroblast growth factor; IGF, insulin-like growth factor; PMN, polymorphonuclear neutrophil; TGF-β, transforming growth factor beta; VEGF, vascular endothelial growth factor; FGF, fibroblast growth factor; HGF, hepatocytes growth factor.

In parallel to the repair of damaged blood vessels, the proliferation and tissue-regeneration phases of healing are characterized by the migration of cells—mainly fibroblasts and keratinocytes. Fibroblasts are drawn from various sources: primarily from the healthy dermis from which fibroblasts can migrate and recruit to the wound; from circulating fibrocytes derived from the BM that have the potential to differentiate into fibroblasts [41]; from BM progenitor cells [42]; and/or from a niche of multipotent precursor cells that reside in the dermis from which they differentiate to dermal fibroblasts [43]. Fibroblasts that are driven from all these sources can be activated and migrated to the wound by factors associated with the damaged tissue, such as hydrogen peroxide (H_2_O_2_), calcium, serum exposure, the loss of mechanical tension, and changes in the electrical gradient [44,45] (Figure 3). The immigration of fibroblasts is also triggered by TGF-β, which builds upon the initial wave that is released by platelets during the hemostasis phase, but at this point, it serves as a pro-mitotic and migration growth factor that helps in the migration of endothelial cells and fibroblasts, allowing for granulation tissue formation (reviewed in [14,46,47]). Activated fibroblasts play a mechanical role by differentiating into myofibroblasts: specialized cells with a contractile ability that help to re-approximate the wound edges, promoting wound closure (Figure 3) [48]. Notably, fibroblasts contribute to the repair of the damaged dermis, mainly by producing collagen, a component of scar tissue, to replace the fibrin-based extracellular matrix (ECM) formed during hemostasis with a collagen-based ECM [46]. This replacement of the ECM is enhanced by the ability of TGF-β to induce the deposition of collagen by fibroblasts and promote fibronectin synthesis [14,49,50]. The response of human fibroblasts to TGF-β depends on the level of TGF-β, as higher concentrations induce cell migration and lower concentrations are better at promoting proliferation [14,51].

The migration of keratinocytes is also evident in this phase. As with fibroblasts, keratinocytes are triggered by stress signals released from damaged tissue, including serum exposure, peroxides, and changes in mechanical tension and the electrical gradient [48,52,53,54]. Activated keratinocytes lie across the granulation tissue for the re-epithelialization process (Figure 3). This process also requires signals from growth factors such as fibroblast growth factor (FGF) and hepatocyte growth factor (HGF), which are secreted from reparative macrophages [7]. Reparative macrophages, which are also referred to as wound-healing macrophages, can release a plethora of growth factors and molecular targets that are associated with collagen production and ECM remodeling. These factors include PDGF, TGF-β, FGF, and resistin-like molecule α (RELM-α), the last of which mediates the production of lysyl hydroxylase (LH), which directs the healing outcome through the formation of a collagenous scar [9,10]. Alternatively, TGF-β negatively regulates the process of re-epithelialization, and a low level of this protein is associated with epidermal hyperproliferation (Figure 3) [55,56]. Nevertheless, the outer epidermal layer of the skin is typically closed after the proliferative phase, and the inside of the wound is still infiltrated by collagen and cellular infiltrates of granulation tissue, so it does not regain the integrity and plasticity of healthy skin.

### 2.4. Tissue-Remodeling Phase of Healing

**Figure 4:**
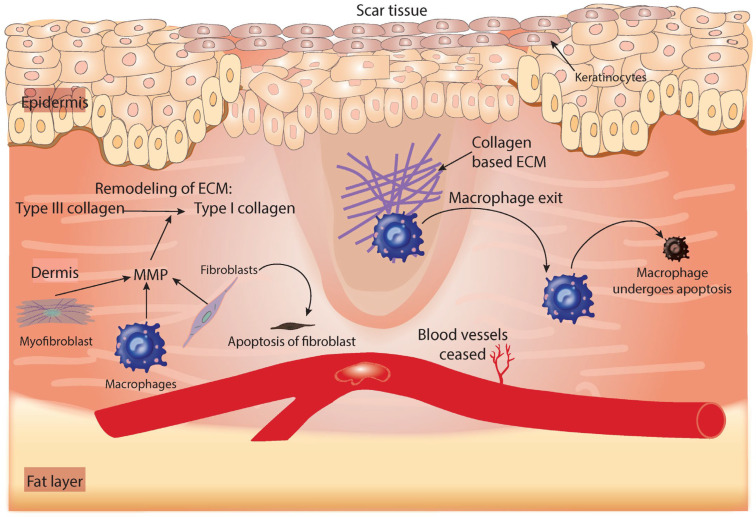
Summary of the process that occurs during the tissue-remodeling phase of healing. The release of MMP from fibroblasts, macrophages, and myofibroblasts helps to remodel the ECM by converting type III collagen into type I collagen. Fibrin-based ECM made during the first phase of healing is replaced by collagen-based ECM. The very few cells that remain in the wound are removed by apoptosis and neovascularization ceases. ECM, extracellular matrix; MMP, matrix metalloproteinase.

After the closure of the wound epidermis, the collagen produced within the dermis is remodeled through a process that balances collagen formation and collagen break-down with the assistance of myofibroblasts differentiated from fibroblasts. The remodeling of collagen occurs in response to the release of matrix metalloproteinase from macrophages, fibroblasts, and epithelial cells [57]. Matrix metalloproteinases convert type III collagen into a stronger form of collagen (type I) (Figure 4) [58]. Cellular components, such as fibroblasts, macrophages, and endothelial cells, are removed from the wound at this stage by apoptosis [59], leaving the wound with a bulk collagen bundle that contains very few cells. Collagen deposition is fine-tuned by the combined effects of TGF-β1 and TGF-β3, which indicates the implications of their exogenous addition in the development of fibrosis and scar tissue formation [14,60,61,62]. As tissue remodeling proceeds, the blood vessel number and blood flow decline, forming an avascular environment (Figure 4). Ideally, remodeling should begin 2–3 weeks post-injury and may take several months. In some conditions, when tissue remodeling is deregulated, excess ECM tissue is formed, and the hypercrosslinking of collagen occurs and extends beyond the wound area into the normal dermis, failing to regress over time and forming a condition known as keloids. Alternatively, and in response to surgery or burns, the skin can generate excess collagen bundles, creating hypertrophic scars that may regress within six months (reviewed in [63]). This overhealing that occurs in the form of hypertrophic scars and keloids is related (non-exclusively) to the excessive production of TGF-β (reviewed in [64]). The challenge of hypertrophic scars is that, when they appear over a tendon or in a critical area, they can be disfiguring or can prevent the normal mobility of a joint [7].

A decade ago, it was generally accepted that inflammation constituted a process that could direct tissue regeneration and determine the outcome of healing, but a further explanation of the mechanisms in this theory was lacking. In a detailed investigation of the role of macrophages induced by the Th2 stimulus IL-4, it was shown that the fate of wounds (regeneration vs. the formation of fibrotic tissue) was directly linked to macrophages [9]. The IL-4Rα downstream target RELM-α (in mice) or RELM-β (in humans) directs the pathway of wound resolution by inducing the production of the LH2 enzyme, which crosslinks collagen bundles to form scar tissue [9].

To summarize, macrophages are not only an essential component of the wound-healing process but also a mechanism that links different phases to each other and directs the healing outcome. Tissue-resident macrophages are responsible for the production of cytokines and chemokines, which cause the earliest neutrophil response at the site of damage. Once neutrophils have completed phagocytosis, they need to be eliminated from the wound to prevent chronic inflammation, which is, again, a process directed by macrophages (efferocytosis) [65]. The removal of dead neutrophils from the system is essential for the wound to switch from inflammation to the reparative mode [65]. In addition, macrophages require apoptotic neutrophils, as well as the Th2 cytokines IL-4 and IL-13, to express anti-inflammatory and reparative proteins such as resistin-like alpha receptor (Retnlα), chitinase-like 3 (Chi3I3), and arginase 1 (Arg1), along with the potential anti-inflammatory proteins fibronectin 1 (Fn1) and eosinophilic cationic protein 2 (Ear2) [66].

## 3. Innate Immune Cells in Wound Healing

### 3.1. Monocytes

Following the influx of neutrophils to a wound, epithelial cells release monocyte chemoattractants, including CCL20 and CCL2, to attract monocytes to the site of tissue damage [67]. Similarly to neutrophils, wound-recruited monocytes clear cellular debris, regulate angiogenesis, and recruit further leukocytes to the wound area. The infiltration of the monocyte (CD45^+^Ly6G^−^CD11b^+^) population has been shown to increase three days after skin injury and revert back to the normal non-injured state at Day 6 after the excisional wound [68]. The expansion of the monocytes in the skin wound is correlated with the expansion of their progenitor in the BM (Lin^−^Sca-1^+^cKit^+^FLK2^−^CD48^+^CD150^−^), also known as the myeloid linage committed multipotent progenitor (MMP3) [68]. Monocyte recruitment in murine wound models is mainly regulated by CCL2 and its receptor, CCR2 [69,70], in addition to CX_3_CL1 and its receptor, CX_3_CR1 [69,70]. With respect to their phenotypes, inflammatory monocytes characterized by Gr1^+^ly6C^hi^—CCR2^+^CX_3_CR1^lo^ in mice or CD14^+^CD16^−^ in humans—are the main population of monocytes recruited to the site of injury [70,71]. Alternatively, monocytes can present as the reparative type that is identified immunophenotypically as CCR2^lo^CX_3_CR1^hi^—Gr1^−^Ly6c^lo^ in mice or CD14^−^CD16^+^ in humans. The knockdown of IL-1R1, which responds to the inflammatory IL-1 signal in skin wounds, impacts monocyte cell expansion, particularly the non-inflammatory subsets (Ly6C^lo^) of monocytes, and is associated with delayed wound closure, according to Barman et al. (2019) [68]. Following their infiltration, monocytes can differentiate into macrophages or dendritic cells. A study of Cx_3_CR1^eGFP^ transgenic mice revealed that the majority of monocytes/macrophages in the wound migrate from the bone marrow and comprise the CX_3_CR1^lo-int^ population. These monocyte-derived macrophages express classical and alternative activation markers when isolated from the bone marrow and are present in vivo during the early and late stages of wounding [72]. By contrast, the more mature macrophages (CX_3_CR1^hi^ population), which have been identified as tissue-resident cells that exhibit an anti-inflammatory state and are associated with the resolution of inflammation, are present as a small population. The failure to mature from Cx_3_CR1^lo-int^ to CXC3r1^hi^ is associated with a presentient inflammatory state in individuals with diabetes [72]. This suggests that, using models of chronic inflammation, it would be interesting to therapeutically target the monocyte-maturation process to treat non-resolved inflammation. Despite their maturation process, monocytes can perform their reparative role via communication with other cells, such as epithelial cells and macrophages, through the secretion of growth factors and cytokines.

### 3.2. Macrophages

Even though the role of macrophages in wound healing has been studied extensively, their role within different types of wounds has remained controversial for a long period of time. Lesions in neonatal mice recruit very few, if any, macrophages but heal efficiently and effectively, even in the absence of macrophages [5]. Similarly, neonatal Pu.1^null^ mice that lack macrophages (as well as neutrophils and B cells) exhibit efficient scarless healing [73]. On the other hand, macrophage-depletion studies have shown that a deficiency of macrophages in adult wounds is detrimental to healing. The first depletion study was performed with a guinea pig model in the 1970s using macrophage anti-sera and glucocorticoids [74]. This study presented the first piece of evidence of impaired healing in the absence of macrophages, including delays in the clearance of erythrocytes, neutrophils, and debris from the wound. The appearance of fibroblasts, the main component of ECM remodeling, was also delayed, and their proliferation rate was slower [74]. However, this study was limited by the fact that glucocorticoids can impair the wound-healing process and may have been involved in the negative repair outcomes observed in these experiments.

Studies involving the selective ablation of macrophages from wounds addressed these previous limitations but also confirmed the essential role of macrophages in healing [10,75,76]. In the past decade, studies have used transgenic mice (LysM-Cre/DTR) containing human diphtheria toxin-sensitive macrophage receptors under the expression of the lysosome M promoter modulated by Cre-recombinase to deplete macrophages prior to wounding [10,75]. The same method was used to deplete macrophages using diphtheria toxin, but this was driven by the *CD11b* promoter [76]. All of these groups discovered that the depletion of macrophages resulted in reduced re-epithelialization, delayed wound contraction, and decreased levels of certain growth factors, including VEGF and TGF-β. As a result, wounds demonstrated impaired neovascularization and granulation tissue formation, as well as a reduction in myofibroblasts, thereby delaying the overall pillars of wound repair [75,76].

In their study of macrophage depletion in a time-restricted pattern, Lucas, et al. [10] significantly clarified the specific role of macrophages during each phase of healing. Their investigation revealed that depletion occurred during the inflammatory phase not only delayed the initial repair process but also affected the late repair response. The authors demonstrated that macrophages recruited during the early stages of healing are the same as those that can transit to the reparative phenotype and produce VEGF and TGF-β, which contribute to wound angiogenesis and myofibroblast differentiation [10]. In comparison, macrophage depletion during the proliferative phase of healing was found to alter the active repair process and increase the apoptosis of endothelial cells. During this stage, macrophages also function in dermal–epidermal interactions, as evidenced by the detachment of the wound epidermis from the underlying granulation tissue in macrophage-depleted wounds [10]. Together, the results of these studies demonstrate that adult wound healing would not be successful without the presence of macrophages and that these cells can exhibit multiple functions during the various stages of healing.

#### 3.2.1. Macrophage Polarization: From Early Discoveries to Recent Updates

The concept of classical macrophage activation was identified in the 1960s, when macrophages in mice showed the first signs of antimicrobial activity in response to infection with Mycobacterium Bovis bacillus (BCG) or Listeria monocytogenes [77]. This concept was extended and developed further by Dalton, et al. [78], who identified the product that activates these macrophages as interferon gamma (IFN-ɣ) from T helper 1 (Th1) cells or natural killer (NK) cells. Indeed, the direct activation of macrophages by the bacterial cell wall product lipopolysaccharide (LPS), alone or together with IFN-ɣ, was identified as the main inducer of classical macrophage activation. In Stein et al. [79], the complexity of the macrophage phenotype was elucidated further through the identification of macrophages produced from IL-4 cytokines as alternatively activated macrophages. By 2003, the concept of alternative macrophage activation was determined to reflect macrophages derived from IL-4 (M2a) or IL-13 exposure. These are distinct from macrophages derived from IL-10—“(M2c) immune suppressor macrophages”—but share some overlapping characteristics, such as the inhibition of inflammatory cytokines and the upregulation of the mannose receptor CD206 [80]. Immune complexes with bacterial lipopolysaccharides or IL-1β stimulate the production of another subtype of alternatively activated macrophages (M2b) [81]. These cells are clearly distinct from both M1 and M2a but share a common expression profile of increased IL-6, IL-1β, and TNF and the sustained production of the anti-inflammatory cytokine IL-10. On the other hand, the M2d subtype of macrophages is proangiogenic, producing high levels of VEGF, TGF-β, and IL-10 [82,83]. Macrophages of this subtype also downregulate the proinflammatory TNF and IL-12 and can be stimulated by IL-6 and adenosine [82]. Whether these subdivisions of M2 macrophages are strictly separated in vivo remains a question that needs further investigation. 

The terms M1 and M2 were initially proposed to describe the two antagonistic pathways of nitrogen metabolism in LPS- or IFN-ɣ-induced macrophages from Th1 murine models (C57BL/6) or Th2 models (BALB/c mice) [84]. Subsequent studies have identified other stimulators of the M1 or M2 phenotype, with the M-CSF-differentiated macrophages showing a phenotype similar to that of the alternatively activated macrophages (upregulating IL-10 but not IL-12) and GM-CSF-derived macrophages showing a phenotype with increased TNF and IL-1, closely resembling the M1-macrophages [85]. 

From a functional point of view, classically activated M1 macrophages promote resistance against microbes, parasites, and tumors by producing high levels of TNF, IL-12, IL-6, reactive oxygen species (ROS), reactive nitrogen intermediates (RNIs), and reactive oxygen intermediates (ROIs) [86]. On the other hand, M2 macrophages promote tissue remodeling, angiogenesis, allergic reactions, and parasite clearance. Although the mode of activation of M2 subtypes can vary, these M2 subsets share some overlapping functions and gene expression profiles. M2 macrophages are often characterized by increased arginase activity and the induction of mannose receptors, scavenger receptors, and TGF-β, in addition to the downregulation of proinflammatory markers, including IL-12, TNF, and nitric oxide synthase (NOS2) [87,88]. Markers that were also used to identify alternatively activated macrophages have been employed in studies on wound macrophage phenotypes. These include found in inflammatory zone 1 (Fizz1), also known as Resistin-like molecule α (RELM-α), and chitinase-like secretory lectin (Chil3), also known as Ym1 [89].

The simple use of classical and alternative macrophages that were used initially to describe the differences in the exogenous stimuli does not accurately reflect the phenotypic changes in vivo. Even the terms M1 and M2, which were assigned to represent differences in arginine metabolism in response to Th1 and Th2 lymphocytes in two different strains of mice [84], do not reflect the macrophage activation status in wounds. The extended categorization of macrophage phenotypes (M2a, M2b, etc.) expanded the definition but described in vivo as a spectrum rather than a defined group. For the above reasons, a common framework of macrophage activation review suggested unified language to describe macrophages polarization, particularly in an experimental setting to either describe them by their collective markers or by defining the activator such as M(IL-4) or M(Inf-ɣ). Alternatively, macrophages can be identified by their source [90]. In this review, we use the terms proinflammatory and anti-inflammatory/reparative when describing macrophage phenotypes in wound healing.

#### 3.2.2. Macrophage Polarization in Wounds

The in vivo classification of macrophages is not as distinct as that in in vitro studies. Within a wound, the macrophage phenotype is best described as a continuous spectrum of various phenotypic states that can change depending on the wound’s microenvironment and the cell’s own intrinsic factors [91,92]. This heterogeneous population within the wound is dominated by proinflammatory macrophages during the early inflammatory phase, and this switches to reparative macrophages during the proliferative and tissue-remodeling phases. However, it is also common to find a population that presents with a mixed phenotypic state that can, for example, express markers of alternative activation, such as CD206, as well as express TNF [91]. The proinflammatory–reparative switch in vivo is certainly associated with normal wound resolution, collagen production, and angiogenesis [91,92]. This is because the reparative macrophages are a rich source of factors such as VEGF and TGF-β [15,28,39,93].

With respect to the stimuli of reparative macrophages, IL-4 and IL-13, which were proposed as central mediators of the alternative activation of macrophages in vitro [80,88,94], gave rise to the reparative phenotype of macrophages in wounds [95]. However, in a sterile wound model, no evidence of the presence of IL-4 or IL-13 cytokines, their receptor IL-4Rα, or their downstream target, pStat6, in wound cells was found [32]. This could be because of the absence of invading microbes, which may alter inflammatory cells and subsequently disturb the pathway that facilitates the switch to M2 reparative macrophages, as it depends on inflammatory cell resolution.

In the past five years, research evidence has challenged the initial view on the absence of the involvement of IL-4 and IL-13 during the in vivo activation of the M2 signature. Models of skin, liver, and lung wounds all clearly demonstrated that knocking down the IL-4Rα signaling in macrophages impaired the healing process in injuries induced by skin punch biopsies, chemical injuries, or helminth infections [9,96,97]. IL-4Rα has also been shown to stimulate the proliferation of tissue-resident macrophages in vivo [98].

Knipper et al. [9] provided important clues about the regulation of M2 macrophages in vivo and demonstrated the existence of functional IL-4Rα in wound macrophages. The conditional depletion of IL-4Rα from whole wounds or from leukocytes using (Il4ra^fl/−^ Lyz2Cre) models with the conditional targeting of IL-4R by inserting the loxp site attenuated the M2 signature in the wound, as evidenced by reduced concentrations of CD163, CD206, and IL-10 and increased NOS. Furthermore, the functionality of the macrophages was impaired as a result of IL-4Rα^−/−^, leading to the hemorrhage of granulation tissue, impaired epithelialization, and the reduced proliferation of macrophages [9].

Signals from Th2 cells and apoptotic neutrophils were originally identified as distinct signals. Indeed, a single treatment of BM-derived macrophages with dead neutrophils alone or with IL-4 or IL-13 was not sufficient to initiate the pro-repair response in helminth infection. Alternatively, co-treatment with Th2 cytokines and apoptotic neutrophils resulted in the dramatic expression of wound-healing genes, including RELM-α, Chil3, Retnlα, and fibronectin 1 (fn1) [66,99]. Molecules that activate anti-inflammatory macrophages can be regulated by microRNA-21 (miR-21), which regulates the efferocytosis-mediated suppression of the innate immune response [99,100]. The mechanism of action of miR-21 is the induction of the silencing of the signaling molecule phosphatase, which contributes to NF-ⱪB-induced inflammation and TNF expression. Together, these studies identify nucleotides as additional factors that regulate macrophage activation. In the above section, several activators involved in macrophage polarization in wound-healing models were discussed. The ability to adopt different phenotypic states makes macrophages an attractive target that can be modified in pathological states, such as in chronic wounds with delayed healing and areas of fibrosis. The following sections discuss the changes that occur in macrophages in the diabetic environment and different therapeutic approaches that are provided for chronic wounds.

## 4. Effect of Macrophage Dysregulation in Wound Healing

### 4.1. Diabetes

To elucidate the impact of diabetes on immune cells, a reflection on the pathogenesis of diabetes and on how hyperglycemia increases the susceptibility to infection and disturbs the immune reaction is required. Type I diabetes (T1D) is an autoimmune disorder in which the insulin-producing cells—the β cells—in the pancreas are destroyed. This destruction involves several immune cells, particularly dendritic cells, macrophages, and T lymphocytes [101]. Individuals can have factors predisposing them to diabetes without having the disease. However, the continuous triggering of the immune system by viral infection or stress promotes the pathology of diabetes. Whether the trigger is stress-induced or virus-mediated, the destruction of β cells occurs via a common pathway that involves the release of the proinflammatory cytokine IFN-ɣ, the apoptosis of β cells, and the release of the β-cell antigen. This ultimately leads to a block in insulin production and a disturbance of immune reactions [101].

Almost 90% of type 2 DM cases are due to insulin resistance. Prediabetic conditions result in an increased glucose level in the blood and are mostly associated with obese, older, or physically inactive patients [102,103]. Pancreatic islet β cells increase their cell mass to produce more insulin in order to compensate for the resistance [104]. Long-term insulin resistance in T2D may lead to macrovascular complications, such as atherosclerosis, as well as microvascular defects, such as nephropathy, neuropathy, and retinopathy [105]. Immunological alterations in type 2 DM patients affect the recruitment and activation of leukocytes, the level of cytokines and chemokines produced, and the occurrence of apoptosis and tissue fibrosis. In this context, we recently found a link between low-grade inflammation in the obese population and the tendency to develop diabetes [106]. Inflammatory cytokines, particularly CCL2, which binds to the CCR2 receptor on monocytes, were found to be elevated in females but not in the obese male population involved in this study [106]. CCL2 regulates the recruitment of monocytes from the circulation to adipose tissue, where they turn into macrophages, particularly the inflammatory M1 type. Early in 2003, it was found that the overexpression of the CC motif chemokine and its ligand, CCL2, were essential for insulin resistance [107]. Alterations in the immune system in diabetes affect the phagocytosis of inflammatory cells, interrupting all the phases of this process, from the extravasation of immune cells from the circulation to the tissue to the ROS production necessary to kill the phagosome [104]. The details of the defective immune system present in individuals with type 2 DM were recently reviewed [104]. Prediabetic patients require an inflammatory trigger to cause the development of type 2 DM. This pathological mechanism involves tissue hypoxia, the NF-ⱪB pathway, IL-6 cytokines (a sensor for insulin resistance), and IL-1, which contributes to the destruction of β cells [104]. Altogether, the above discussion shows that immune cells are not only affected by diabetes but are also involved in the pathogenesis and destruction of pancreatic β cells in type 1 DM and in inflammation in prediabetic obese patients who are prone to the development of type II DM.

### 4.2. In Diabetic Models

The dysregulation of myeloid cells, whether in diabetic patients or mouse models, affects their maturation, phenotypic states, and function. Several reports on diabetic macrophage phenotypes in models mimicking type 2 DM described them as macrophages with a sustained inflammatory profile that ineffectively switches to the reparative mode needed for collagen production, angiogenesis, and growth-factor production [91,108]. The investigation of proinflammatory and anti-inflammatory/reparative macrophages from leptin-receptor-deficient^(db/db)^ murine models, mimicking type 2 DM, demonstrated that, while nondiabetic-derived macrophages can effectively express the anti-inflammatory profile later in healing, diabetic cells continue to display a sustained proinflammatory profile, demonstrated by increased matrix metalloproteases such as (MMP)-9, iNOS, and reduced IGF and TGF-β until Day 10 after wounding [92]. The presence of M1-inflammatory cells in the wound until the late stages of healing can negatively impact the healing outcome, causing diabetic wounds to remain in an inflammatory phase and inhibiting their progression to the proliferation and tissue-regeneration phases of repair.

Skin biopsies from diabetic foot ulcers have shown that a failure to induce M2 macrophages in diabetic patients can differentiate healing from nonhealing ulcers. Bannon, et al. [91] examined the dual expression of CD68 (macrophage marker) and either Arg1^+^ (M2-reparative marker) or Nos2^+^ (M1-proinflammtory marker) in skin biopsies extracted from foot ulcers of patients who received best patient care. The authors found that, in nonhealing ulcers examined over a period of 6 weeks, the CD68^+^Arg1^+^ population was significantly reduced in comparison with that in healing ulcers. By contrast, the occurrence of the CD68^+^Nos2^+^ population did not differ between nonhealing and healing diabetic foot ulcers. These data confirm that a failure to induce the M2 phenotype specifically of macrophages in human diabetic foot ulcers is a poor prognostic factor for ulcer healing in type II diabetic patients [91].

Under in vitro conditions, macrophages derived from diabetic patients are hypersensitive to classical and alternative activation and produce extreme forms of inflammatory and anti-inflammatory macrophages when stimulated by LPS or anti-IFN-ɣ antibodies [91]. In a model of type II diabetes, the defective macrophage phenotype shown was described as having intrinsic changes within cells caused by diabetes. These changes began as early as cells matured in the BM and appeared even when diabetic cells were transplanted into nondiabetic mice [91].

Streptozotocin-induced type 1 diabetic rat models displayed a different macrophage polarization defect pattern that led to impaired wound repair [108]. Wound macrophages in type 1 db rats were shown to have an insufficient proinflammatory profile early in healing that failed to meet the wound’s demand during the inflammatory phase of healing. This was followed by the development of an extensive anti-inflammatory profile later in proliferation and tissue regeneration phase [108]. Interestingly, one would think that, in these models, the wound would heal perfectly, as the pattern opposes the defects observed in db/db mice with type II DM. However, these models also failed to show successful healing, as was observed with type II diabetic murine models. These observations support the theory that any impairment of the macrophage polarization profile—whether through excessive inflammation, as in type II models, or through a reduction in inflammatory macrophages, as in type 1 models—will yield the same negative outcome of impaired healing and may correspond to the presence of ulcers in diabetic patients.

A population of CCR2^+^ macrophages recruited at an early time point in normal wounds exhibited markers from both inflammatory and reparative macrophages as well as expressing VEGF-A at the site of skin injury that promotes neovascularization [38]. This was confirmed using CCR2-eGFP reporter mouse models combined with an analysis of mutant mice myeloid cells restricted to CCR2 or VEGF signaling [91]. However, these mixed-polarized macrophages expressing both proinflammatory and reparative markers are retained in diabetic wounds but failed to stimulate angiogenesis [91]. This confirms the impact of diabetes on the function of macrophages. To explain these defects, one could argue that diabetic macrophages and myeloid cells are intrinsically defected, therefore, cannot perform functions such as angiogenesis. This intrinsic defect observed in their maturation process, as shown by reductions in the F4/80 and CD11b maturation markers in macrophages isolated from the BM, confirms that this problem originates in the BM rather than as a result of the wound environment alone [91].

Studies on sterile wound models or polyvinyl alcohol sponge-derived macrophages have provided some answers regarding the maturation state of macrophages in relation to their phenotype [32,109]. During the early stages of healing, the F4/80^+^Ly6C^hi^ inflammatory monocyte population can either remain without any further differentiation or rapidly transform into proinflammatory monocytes by acquiring markers such as CD14 and TNF [109]. As healing proceeds to the mid-stage, macrophages derived from F4/80^+^Ly6C^lo^, the “more mature population”, begin to acquire other surface markers, such as the co-expression of CD64 and MerTK along with CD206 [109]. These Ly6C^lo^MertK^+^CD64^+^ cells release pro-repair mediators, such as VEGF and TGF-β, suggesting that the acquisition of the reparative phenotype in wounds is accompanied by an increase in the macrophage maturation potential [109]. Conversely, the proinflammatory phenotype is associated with a less mature population of cells that express Ly6C^hi^ [109]. Similarly, macrophages in the gut showed alternative activation in response to nematode infection, as assessed by the expression of RELM-α in populations identified as resident macrophages expressing CX_3_CR1^hi^F4/80^+^Ly6C^−^. However, this was not shown in an immature population of Ly6C^+^ monocytes [110]. Thus, both skin wound and intestinal infection models support the hypothesis that the macrophage phenotype—notably, the reparative type—is associated with the more mature developmental stage and with the resident rather than the recruited population.

Based on these associations, diabetic wound macrophages that fail to mature normally, as described in numerous studies [91,92,108], are probably the same cells that fail to polarize to the reparative fate associated with increased inflammatory cellular infiltration and chronic wound pathologies. Consistent with the maturation defect in murine macrophages reported by Crane et al. [109], inflammatory macrophages derived from human patients with diabetes were also found to be less mature, as determined by the maturation transcription factor Runt-related transcription factor 1 (RUNX1), the ETS family transcription factor SPI1/PU.1, and the CAAT enhancer-binding protein alpha (CEBP-α) [111]. The transcriptional relationships among RUNX1, SPI1, and CEBP-α in relation to the maturation of myeloid cells have been discussed extensively in the literature (reviewed in [112]). The aberrant expression of endogenous myeloid transcription factors represents one mechanism that can explain the diabetic phenotype of persistent immaturity. A diminished level of the key myeloid transcription factor that regulates maturation, CEBP-α, was found to result in monocyte and granulocyte immaturity in diabetic cells [113]. The role of the CEBP-α and SPI1 transcription factors is not restricted to maturation, as these factors also promote the recruitment of the NF-ⱪB subunit p65 at the enhancer sites and regulate cell activation by LPS [114].

Investigating the deregulated pathways in myeloid cells isolated from the BM of diabetic mice and from the site of injury showed that the NF-ⱪB and JAK/STAT pathways are the main defective pathways that cause chronic inflammation in diabetes, whether at the wound site or from the BM early in development, confirming that intrinsic factors play roles in the formation of defective macrophage phenotypes [115]. The relationship between the microenvironment and intrinsic cell factors in diabetic myeloid cells was recently studied [115]. In a histological sample extracted from a Day 3 diabetic wound, the expression of the epigenetic regulator RelA, a member of the class II NF-ⱪB family, was not affected by the diabetic environment, and its phosphorylation potential was not changed. However, the RelA inhibitor IⱪBα was shown to be downregulated at the mRNA and protein levels. These data were provided from RNA-seq and Western blot analysis using a purified population of Gr1^+^ cells that were identified to be early myeloid cell responders at the injured site [114]. RelA and Stat3 act synergistically on the promoter region of the inflammatory gene and the chromatin-modifying enzyme p300 to acetylase their loci, thereby promoting their expression [116,117]. The downstream target of the NF-ⱪB pathway, IFN-ɣ, is a potent activator of tissue macrophages that regulates Stat1 expression, inducing a state of protection against foreign pathogens in the affected region [118]. Stat1 and Stat3 are clearly overactivated in a diabetic environment, which explains many of the upregulated inflammatory cytokines [119]. Altogether, these data suggest that the sustained inflammatory response observed in diabetic mice as well as the persistent immaturity may result from the aberrant regulation of epigenetic factors, such as chromatin-remodeling enzymes, in diabetic myeloid progenitor cells.

It has become clear over the past two decades that the diabetic environment reduces the reparative state and amplifies the proinflammatory signature, creating a state of continuous inflammation and poor wound healing. Evidence from in vivo and in vitro studies shows that changes in the macrophage phenotype are mainly the result of hyperglycemia, highlighting the importance of diabetic patients controlling their blood sugar levels [120]. A hyperglycemic microenvironment was shown to increase the M1:M2 ratio, as determined by the level of CCR2, an M1 marker, in relation to the level of CD48, an M2 marker, found in the perilesional dermis of db/db mice [120,121]. The increase in the M1:M2 ratio due to hyperglycemia was confirmed to also occur in humans by the same group through an in vitro study. The increase in proinflammatory macrophages led to increases in MMP1 and TNF production, both of which impaired keratinocyte migration and delayed the re-epithelization process involved in wound repair [121]. Morey et al. [122] found that about 13 inflammatory cytokines were elevated due to hyperglycemia and the hypoxic microenvironment, including TNF, IL-1, and IL-12. This diabetic microenvironment effect was represented as a synergy between hyperglycemia and hypoxia, which created a continuous trigger for proinflammatory cytokines [122]. The diabetic microenvironment impacted the process of cell migration to the wound, with fewer progenitor cells being recruited to the wound and more inflammatory cells migrating to, or being retained in, the injured sites. The overproduction of MCP-1 and MIP-2 chemokines stimulated leukocyte migration in the wound. Neutralizing antibodies against these chemokines were confirmed to contribute positively to the recruitment of neutrophils and macrophages to injured tissue [123]. On the other hand, reparative growth factors, such as TGF-β, VEGF, and FGF, which helped in tissue regeneration and collagen production, were affected and reduced in concentration, which increased the time taken for the wound to heal [124,125]. The above examples represent how the diabetic microenvironment in the wound, such as the high blood glucose level or low oxygen level, impacts the chemokine and cell migration, cytokines and reepithelization. These processes can all be linked to the disruption of the M1 and M2 macrophages ratio as result of hyperglycemia.

#### Epigenetic Changes in Dysregulated Macrophages in Diabetes

The aberrant expression of genes encoding the myeloid cells’ development and the inflammatory signature of macrophages mediated the “less mature-more inflammatory-fate” of macrophages in diabetes. As epigenetic changes in chromatin modifications in macrophages, such as histone methylation, affect gene accessibility, macrophages’ maturation and polarization potential are controlled partially through these chromatin-modifying enzymes. Depending on the site of histone methylation, the end product is either to activate the transcription of the target gene or to suppress its expression [126]. The methylation of lysine (K4) on histone (H3) or H3K4me3 keeps the chromatin in a conformation that enables the promoter for transcription, thereby allowing for the activation of the target genes. One member of the methyl transferase protein that is known to methylate histone on lysine is mixed-lineage leukemia (MLL). It promotes the expression of genes in the NF-ⱪB pathway, thereby impacting the inflammation process during diabetes [127,128,129]. An upstream receptor of the signaling pathway is TLR4, a receptor on the surface of macrophages for LPS, which is a component found on the wall of gram-negative bacteria that is commonly observed in contaminated chronic wounds [130]. Diabetic macrophages demonstrate an increase in the MLL-mediated H3K4me3 of the TLR4 promoter, resulting in increased TLR4 expression. Such findings have been achieved using whole-wound punch biopsies and confirmed by MLL knockout mouse models (MLL^f/f^ Lyz^Cre+2^) [131]. MLL-mediated methylation actively transcribes TLR4, thereby increasing the TLR4 signaling pathway and upregulating the proinflammatory mediators IL1β and TNFα [131]. The data above explain one mechanism by which macrophages remain in a state of constant inflammation through DNA methylation and may open the door to the possibility of TLR4 pharmacological inhibition to contain inflammatory myeloid cells in diabetes. Another histone methyltransferase involved in the epigenetic regulation of macrophages is Setdb2, which works by trimethylating lysine K9 on histone 3 (H3K9me3), resulting in a chromatin conformation that renders the binding site for NF-ⱪB on pro-inflammatory cytokines inaccessible and negatively regulates inflammation. The role of Setdb2 in myeloid cells is further confirmed using myeloid-specific Setdb2 knockout models (Setdb^f/f^/Lyz^Cre+2^) that lack Setdb2 in their monocytes and macrophages [132]. The skin wounds in these models exhibited a significant delay in wound healing, particularly a prolonged inflammatory phase, confirming the control of the Setdb methyltransferase of inflammation. Macrophages isolated from Setdb ^f/f^/Lyz ^Cre+2^ models demonstrated a significant increase in the inflammatory proteins MIP-2, IL1, and IL6 and other NF-ⱪB-mediated cytokines and chemokines, thus confirming Setdb2’s epigenetic regulatory control of inflammatory macrophages and their consequence on inflammation phase of wound healing. Chromatin immunoprecipitation (ChIP) analysis performed on macrophages isolated at Day 5 post-injury in (Setdb^f/f^/Lyz^Cre+2^) mice exhibited a reduction in the methylation of H3K9me3 at NF-ⱪB binding sites in the promoters of inflammatory genes (IL1β, TNFα, and Nos2) [132]. The reduced Setdb2-mediated methylation in that model was accompanied by increased inflammatory proteins at Day 5, a key timepoint at which macrophages are transformed from an inflammatory to a reparative state. Therefore, Setdb2 DNA methylation is a possible regulator of normal macrophages’ phenotypic switching from M1-inflammatory to M2-reparative during wound healing by limiting the inflammatory macrophage signature. Setdb2 controls the functions of macrophages via INF-I through the Jak/Stat1 axis, thereby confirming how cytokines regulate the phenotypes of macrophages through epigenetic changes [133]. In pre-diabetic mice that were induced by a high-fat diet that mimics the state of type II diabetes in humans, the INF-I-Setdb2 axis is impaired, thereby reducing Setdb2 and the trimethylation of lysine (H3K9me3). This activates the transcription of NF-ⱪB binding sites on inflammatory cytokine promoters such as IL-1β, promoting chronic inflammatory phenotypes in these mouse models. These findings are in line with the hypothesis of Jaenisch and Bird (2003) in that environmental changes, such as hyperglycemia or a high-fat diet, can impact the epigenetic changes that occur due to diseases such as cancer and diabetes (reviewed in [126,134]). 

To summarize, the diabetic phenotype of macrophages from type II diabetes in human and murine models represents cells with a prolonged proinflammatory signature that continues until the late stages of repair. These cells demonstrate ineffective switching to the reparative fate that promotes angiogenesis, tissue regeneration, and wound closure. Epigenetic regulators such as MLL and setdb2 mediate conformational changes to the chromatin, rendering the binding sites of inflammatory genes on NF-ⱪB accessible or inaccessible to control inflammation and the macrophages’ fate. Together, this reveals that epigenetic regulators that control the normal patterns of polarization are critical to wound resolution.

## 5. Therapeutic Manipulation of Diabetic Macrophages

Despite being clinically and molecularly diverse, chronic wounds are generally defined as full-thickness cutaneous wounds that fail to heal within three months. These are generally associated with three major clinical complications: leg ulcers, diabetic foot ulcers, and pressure ulcers [6]. The main concern with chronic wounds, whether in human or murine models, is that they fail to close within the expected timeframe, remaining in an inflammatory phase.

The best way to promote the progression of the healing process towards the following phase of healing is through treatment to remove the excess inflammatory leukocytes and to induce re-epithelialization and angiogenesis.

One promising approach for the treatment of chronic wounds is the stimulation or reprogramming of macrophages, considering that cell-intrinsic factors play important role in the deregulation of macrophages’ maturity and phenotype.

Macrophages cross-communicate with other cell types, such as skin epithelium and platelets [70,135]. Cell-based therapy utilizes this powerful communication across the cells during healing. One example would be platelet-rich plasma (PRP) to enhance murine-injured tendon healing using macrophages [135]. In a previous study, the use of the B6.12P-Cx3cr^tm1litt/j^ murine model allowed for the trafficking of macrophages via GFP expression under the control of Cx_3_cr1. Using this model, it was demonstrated that the administration of PRP increased macrophage recruitment in host mice, as measured by CD11b^+^F4/80^+^GFP^+^ cells, allowing for the precise determination of whether the recruitment came from the host mice. The phenotype of the recruited GFP^+^ macrophages (proinflammatory versus reparative) was determined by sorting Ly6C^+^ and CX_3_CR1^+^ cells, respectively. The use of PRP provoked the recruitment of proinflammatory macrophages at an earlier time point in response to PRP treatment, irrespective of the quality of PRP used (leukocyte-rich (LR) or leukocyte-poor (LP)), while the anti-inflammatory macrophages were more prominently infiltrated only in response to treatment with LP-PRP [135]. That study supported the hypothesis that the cytokines and growth factors released from platelets activate the tissue-repair process via a mechanism involving macrophage recruitment, activation, and phenotypic change. We also learned from this study that, in the absence of leukocytes, PRP promotes the M2 phenotype and better healing in murine patellar tendons. Favoring the M2 phenotype of macrophages could serve as a possible therapeutic target in different models of wound healing [111,136,137,138]. The administration of IL-33 directly to wounds in streptozotocin-induced diabetic mice was shown to favor the development of the M2(IL-13)/M2a type, inducing reparative macrophage polarization and its downstream effects, such as improved angiogenesis and increased ECM deposition, as well as a better healing process [137]. 

Another approach in the therapeutic manipulation of wound cells or innate immune cells is the use of transcription factors and intrinsic proteins that are deficient in diabetic macrophages to promote wound healing. One opportunity might involve the utilization of the endogenous anti-inflammatory peptide chemerin 15 (C15), which is administered topically in dorsal skin murine wounds [138]. In a previous experiment, C15-treated wounds showed accelerated healing. Specifically, in collagen, deposition and macrophage recruitment were restricted to the injured site. Essentially, the administration of the inflammatory resolution mediator C15 reprogrammed the macrophage phenotype to produce more arginase- and fewer iNOS-producing TNF-positive cells [138]. One would assume that a more direct application of M2-polarized macrophages into a model of chronic wounds would be a desirable next step in terms of cell-based therapy. Interestingly, the direct administration of M2-polarized macrophages to murine models or diabetic wounds has not been shown to impact the healing time or quality [139]. It is possible that the addition of M2-polarized macrophages earlier in the healing process is not the most suitable application and, in fact, may retard the regulated rhythm of healing or even negatively impact the healing outcome. It is therefore suggested that the therapeutic approach should address both aspects of dysregulated macrophage phenotypes in diabetes: chronic M1-inflammatory stimulation and an attenuated M2-reparative fate.

### Therapeutic Use of Homeobox a3

Homeobox a3 (Hoxa3) is a member of a group of endogenous transcription factors present in normal wounds but reduced in diabetic wounds [140]. The gene transfer of Hoxa3 into the wounds of Lepr^db−/−^ diabetic mice showed accelerated healing compared with a heterozygous control group (lepr^db+/−^), induced the production of neovascularization-associated genes, and suppressed NF-ⱪB inflammatory pathway genes [140,141]. The introduction of Hoxa3 into target cells progressed from the gene transfer method to the protein transduction technique by utilizing an endogenous structure that is present in all homeobox proteins (i.e., pentratin peptide), which allows the protein to be introduced into target cells without the need for endocytosis or receptors for entry [111,136,142,143,144]. The transduced proteins were first used as feeder cells that were transfected with Hoxa3 using the long-term co-culture method, and Hoxa3 was introduced into HSC/progenitor cells (target cells) via passive translocation [144]. The Hoxa3 protein overexpression induced by this method positively reprogramed HSCs by reducing their multipotency and allowing for their maturation into CD11b^+^Ly6G^+^ cells. CD11b^+^Ly6G^+^-Hoxa3-induced cells were able to migrate toward the macrophage inflammatory protein (MIP-1) and the MCP-1 chemoattractant and reduce their adhesion to TNF-activated endothelial cells, thereby confirming the migratory maturation role of Hoxa3 [144]. The protein transduction method was then modified to include overexpression using a condition medium enriched with Hoxa3, where the protein product was easily transferred from the transfected cell into the medium by a secretory protein and from the medium into the target cells using a penetratin peptide [136,142,143,145]. The use of the Hoxa3-enriched medium on BM-derived macrophages resolved the imbalance between the proinflammatory and reparative phenotypes of diabetic macrophages and suppressed the production of inflammatory cytokines, such as TNF and nitric oxide, from proinflammatory macrophages. Nevertheless, the augmentation of the *Mrc1* and *Chil3* reparative markers of reparative macrophages, as well as the suppression of the *Ccl2*, *CD68*, and *Tnf* inflammatory markers, was shown, thus confirming the anti-inflammatory reparative properties of Hoxa3 for ex vivo BM-derived macrophages [136]. The reductions in the expression of the *CCL2* and *TNF* genes as well as the *TNF* promoter in Hoxa3-treated human diabetic macrophages in recent reports confirm that some of Hoxa3’s effects can be translated to human cells. In that experiment, Hoxa3 was introduced as a purified recombinant protein that could passively translocate to monocyte-derived macrophages. With progression in the technique used to introduce Hoxa3, the results obtained supported the same hypothesis of the prohealing anti-inflammatory protein. A possible mechanism by which Hoxa3 may limit inflammation was investigated in that experiment by modulating the p65 subunit of NF-ⱪB and investigating its association with the aberrant inflammatory phenotype seen in diabetes [111].

Inflammatory macrophages are associated with markers of immaturity, as reported by Bannon et al. [91], Crane et al. [109], and many others. The impact of Hoxa3 on the maturation process was examined, and it was found that, in BM-derived murine macrophages, the presence of the Hoxa3 protein increased the F4/80 and CSF1R/CD115 flow cytometer maturation markers [136]. Therefore, Hoxa3 can rescue both the defective phenotype and persistent immaturity; this is not surprising, as, in diabetes, the persistent inflammatory phenotype and immaturity of myeloid cells are intertwined processes. The importance of CSF1R in macrophage maturation lies in its role as the macrophage colony stimulating factor (MCSF) receptor as well as it being the target of a synergistic effect caused by the transcriptional machinery of Spi1, Runx1, and Cebpα (reviewed in [112]). The regulator of MCSFr, the Spi1 protein level, and mRNA were found to be augmented in response to Hoxa3 protein treatment in murine macrophages [136]. In an investigation of whether Hoxa3 can rescue the deregulated transcription factors in human cells, it was shown that, in monocyte-derived macrophages from type II DM patients, the levels of Spi1 and Runx1 were upregulated, confirming the changes observed in mouse macrophages and the rescue of cells’ immaturity [111]. This means that the induction of some maturation markers in diabetic macrophages by Hoxa3 treatment could be partly achieved by modulating a pathway involving the Spi1 and/or Runx1 transcription factors. The mechanism underlying the effect of Hoxa3 on the promotion of the reparative macrophage phenotype may involve increased Stat6 protein and Stat6 phosphorylation. Stat6 undergoes phosphorylation and dimerization processes before it translocates into the nucleus of target cells and upregulates the reparative genes. We found that, in response to Hoxa3 overexpression, there was an increase in pStat6 concentration, explaining the mechanism by which Hoxa3 mediated the reprogramming to the reparative fate [136].

Moreover, when Hoxa3 was injected into wounds on diabetic mice, the population of Nos2^−^Arg1^+^ (reparative) cells, along with that of the Mac3^+^ (macrophage marker) VEGF^+^ (proangiogenic marker), increased on Day 7, revealing that Hoxa3 promoted reparative macrophages not only in ex vivo-derived macrophages from humans and mice but also in in vivo wounds [136].

In summary, the studies outlined above demonstrate that targeting the intrinsic defects in macrophage maturation and phenotypes promotes therapeutic manipulation of macrophages that leads to improved wound healing. The main point of several examined papers was that, through the safe manipulation of macrophages’ phenotypes, the healing could be accelerated, and their quality is improved. Cross-communication among innate immune cells, epithelial cells, and cytokines played an undeniable part in such an outcome. Whether administrating PRP, intrinsic factor, or regulatory proteins, all showed positive examples of macrophage reprogramming or phenotypic changing from inflammatory to reparative. However, a deeper understanding of the underlying mechanism of macrophage reprogramming needs to be achieved in the future before translational studies in human clinical trials are conducted.

## 6. Concluding Remarks

It is clear that macrophages play an important role in wound healing, and the regulation of their activation state is key to each stage in this process. Macrophages in a wound are best described as being in a mixed polarized state dominated by proinflammatory cells before being transformed to a state dominated by reparative/anti-inflammatory cells. This parallels the transition that occurs between stages in the injury-repair process. Nevertheless, questions remain regarding the details of the maturation process for macrophages in diabetes and the interplay of this with the phenotype and function. More thorough in vivo studies should be conducted to gain a better understanding of the maturation process for macrophages and how it is related to the persistent inflammatory state observed in diabetic wounds. Another challenge in this field is the identification of the exact subtypes of macrophages and the inconsistencies between models used in in vitro and in vivo studies.

In 2010, Lucas et al. [10] developed a LysMCre/iDTR murine model that allowed for the conditional deletion of macrophages at different phases of healing. Depletion in the early stages reduced vascularization and delayed the healing time, while that in the mid-stages resulted in severe hemorrhaging in wound tissue. However, there was no significant impact on the outcome of healing when macrophages were depleted in the late stages of repair. The depletion of macrophages was also achieved via the administration of an agonist to inhibit one of several components of the signaling pathway of macrophages, such as the method used by Klinkert et al. [146] in 2017. In that study, M2 macrophages were inhibited using GW2580, an inhibitor of CSF1 signaling that regulates the proliferation and differentiation of M2/reparative macrophages. To investigate their effect, macrophage Fas-induced apoptosis (MaFIA) transgenic mice with a macrophage-specific promoter used to express green fluorescent protein (GFP) were used to allow for cell tracking. M2-macrophage depletion resulted in delayed healing, persistent inflammation, the accumulation of neutrophils, and the attenuation of collagen deposition in models of sterile surgical wounds [146]. In contrast to the common dogma, as shown back in 2003, PU.1^−/−^ neonatal transgenic mice showed perfect healing with no scars when both neutrophils and macrophages were depleted [73]. A comparison of different healing phenotypes in wound models of depleted macrophages was discussed recently [147]. This study focused on how the skin healed in each model; whether the stem cells, fibroblasts, or epithelial cells potentially replaced the lost macrophages; and whether this depletion impacted scar formation [147].

Sirtuin 6 is an NAD-dependent histone deacetylase that is involved in transcriptional silencing and is localized in the nucleus [148]. Recently, the mS6KO murine model, in which Sitrt6^flox/flox^ mice breed with LysMCre, was used to investigate the impacts of Sirt6 on macrophage migration, inflammation, and wound repair [149]. The absence of Sirt6 delayed wound closure in comparison to the wild type, and the infiltration of macrophages increased, but macrophages failed to switch to the M2 reparative fate, favoring the M1 signature. Increased macrophage infiltration was accompanied by the production of the CCL2 chemoattractant, but STAT6 phosphorylation, which regulates M2 (IL-4) macrophages, was not affected. This shows that Sirt6 plays a role in the macrophage phenotypic switch and thus affects the outcome of healing. There are numerous examples of the effective use of different Cre-based models, particularly in the areas of macrophages and wound healing. The very first example of inducible macrophage depletion using LysMCre/DTR was applied by Goren and colleagues [75] in 2009. This method, together with many other models of macrophage depletion, highlights that the absence of macrophages is detrimental to the healing process, whether stage-specific [10]; specific for the M2 phenotype, as in anti-CSF mediated inhibition [146]; or from targeting the epigenetic regulator of macrophages, as with Sirt6 [149]. This led to a focus on therapeutic-based research to investigate the targeting of macrophages as a desirable approach for chronic wound treatment, especially in models in which macrophages can play different roles during the healing process. By elucidating the overexpression of genes or protein products that are deficient in diabetic wounds, it has been made clear that macrophages can be reprogramed to function similarly to normal ones, as observed with Hoxa3 gene delivery to wounds and protein delivery to cells isolated from diabetic mice [111,136,140,141]. Hoxa3-treated macrophages exhibit the attenuation of the chronic proinflammatory fate (which retards wound progression) and the augmentation of the reparative fate (which controls the stages of proliferation and regeneration), with Hoxa3 alone being able to accelerate the healing of chronic wounds. However, it remains unclear whether the addition of Hoxa3-treated macrophages would constitute an ideal wound treatment, as adding reparative macrophages to chronic wounds has not been shown to improve the healing outcome [139]. The timing of delivery and the conditions required to optimize the delivery of the Hoxa3 protein into a wound need to be carefully designed. It is also important to determine whether the targeting of macrophages alone is sufficient for healing. An improved understanding of all the key factors that acted together to create the impaired healing state will ultimately lead to advancements in healing therapies.

## Data Availability

Not applicable.

## Abbreviations

Arg1: arginase 1; BM, bone marrow; C15, chemerin 15; Ccl2, chemokine C-C motif ligand; CEPB-α, CCAAT-enhancer-binding protein alpha; Chi3I3, chitinase-like 3; Chil3, chitinase-like secretory lectin; DM, diabetes mellitus; Ear2, eosinophilic cationic protein 2; ECM, extracellular matrix; FGF, fibroblast growth factor; Fizz, found in inflammatory zone 1; Fn1, fibronectin 1; HIF-1α, hypoxia inducible factor; HMGB, high-mobility group box 1 protein; HSC, hematopoietic stem cell; HSC/P, hematopoietic stem cell/progenitor; IGF, insulin-like growth factor; IL, interleukin; IFN-ɣ, interferon gamma; iNOS, inducible nitric oxide synthase; LP, leukocyte poor; LPS, lipopolysaccharide; LR, leukocyte rich; MCP, monocyte chemotactic protein; MIP, macrophage inflammatory protein; MMP, matrix metalloproteinase; NF-κB, nuclear factor kappa B; NOS, nitric oxide synthase; PDGF, platelet-derived growth factor; PKC, protein kinase C; PMN, polymorphonuclear neutrophil; PPP, pentose phosphate pathway; PRP, platelet-rich plasma; RELM-α, resistin-like molecule; Retnlα, resistin-like alpha receptor; ROS, reactive oxygen species; RUNX1, runt-related transcription factor 1; SCL, stem-cell leukemia; TCA, tricarboxylic acid cycle; TGF-β, transforming growth factor beta; TNF, tumor necrosis factor; VEGF, vascular endothelial growth factor; α-SMA, α-smooth muscle actin; Arg1, arginase 1; IL-4Rα, interleukin 4 receptor alpha; JAK, Janus kinase; Stat6, signal transducer and activator of transcription 6; ECM, extracellular matrix; MMP, matrix metalloproteinase; BM, bone marrow; FGF, fibroblast growth factor; IGF, insulin-like growth factor; TGF-β, transforming growth factor beta; ATP, adenosine triphosphate; DAMP, damage-associated molecular pattern ChIP, chromatin immunoprecipitation; TLR, Toll-like receptors; HSP, heat shock protein; MAMP, microbes-associated molecular pattern; FGF, fibroblast growth factor; HIF, hypoxia-inducible factor; CBP/p300, core-binding factor/protein 300; HRE, hypoxia response element.
